# CME ACTIVITY: Extrapulmonary Infections Associated with Nontuberculous Mycobacteria in Immunocompetent Persons

**DOI:** 10.3201/eid1509.070616

**Published:** 2009-09

MedscapeCME is pleased to provide online continuing medical education (CME) for this journal article, allowing clinicians the opportunity to earn CME credit. This activity has been planned and implemented in accordance with the Essential Areas and policies of the Accreditation Council for Continuing Medical Education through the joint sponsorship of MedscapeCME and Emerging Infectious Diseases. MedscapeCME is accredited by the Accreditation Council for Continuing Medical Education (ACCME) to provide continuing medical education for physicians. MedscapeCME designates this educational activity for a maximum of 0.75 *AMA PRA Category 1 Credits*™. Physicians should only claim credit commensurate with the extent of their participation in the activity. All other clinicians completing this activity will be issued a certificate of participation. To participate in this journal CME activity: (1) review the learning objectives and author disclosures; (2) study the education content; (3) take the post-test and/or complete the evaluation at **http://www.medscape.com/cme/eid**; (4) view/print certificate.

## Learning Objectives

Upon completion of this activity, participants will be able to:

Diagnose and treat nontuberculous mycobacterial (NTM) lymphadenitis effectively
Identify elements of NTM osteoarticular infections
Treat NTM skin infections according to standards of care
Describe infections with rapidly growing mycobacteria

## Editor

**P. Lynne Stockton, VMD, MS,** Copyeditor, *Emerging Infectious Diseases. Disclosure: P. Lynne Stockton, VMD, MS, has disclosed no relevant financial relationships.*

## CME AUTHOR

**Charles P. Vega, MD, FAAFP,** Associate Professor; Residency Director, Department of Family Medicine, University of California, Irvine. *Disclosure: Charles P. Vega, MD, FAAFP, has disclosed no relevant financial relationships.*

## AUTHORS

Disclosures: **Claudio Piersimoni, MD;** and **Claudio Scarparo, MD,** have disclosed no relevant financial relationships.

## Earning CME Credit

To obtain credit, you should first read the journal article. After reading the article, you should be able to answer the following, related, multiple-choice questions. To complete the questions and earn continuing medical education (CME) credit, please go to **http://www.medscape.com/cme/eid**. Credit cannot be obtained for tests completed on paper, although you may use the worksheet below to keep a record of your answers. You must be a registered user on Medscape.com. If you are not registered on Medscape.com, please click on the New Users: Free Registration link on the left hand side of the website to register. Only one answer is correct for each question. Once you successfully answer all post-test questions you will be able to view and/or print your certificate. For questions regarding the content of this activity, contact the accredited provider, CME@medscape.net. For technical assistance, contact CME@webmd.net. American Medical Association’s Physician’s Recognition Award (AMA PRA) credits are accepted in the US as evidence of participation in CME activities. For further information on this award, please refer to http://www.ama-assn.org/ama/pub/category/2922.html. The AMA has determined that physicians not licensed in the US who participate in this CME activity are eligible for *AMA PRA Category 1 Credits*™. Through agreements that the AMA has made with agencies in some countries, AMA PRA credit is acceptable as evidence of participation in CME activities. If you are not licensed in the US and want to obtain an AMA PRA CME credit, please complete the questions online, print the certificate and present it to your national medical association.

### Article Title: Extrapulmonary Infections Associated with Nontuberculous Mycobacteria in Immunocompetent Persons

## CME Questions

1. Which of the following statements about nontuberculous mycobacterial (NTM) lymphadenitis is most accurate?
  A. It is the most common NTM disease in children
  B. The most frequently isolated organism is *Mycobacterium haemophilum*
  C. NTM adenitis almost always presents with the sudden onset of severe illness
  D. Chemotherapy is more effective than surgical excision for uncomplicated NTM lymphadenitis
2. Which of the following statements about osteoarticular infections with NTM is most accurate?
  A. NTM are usually spread through the blood to the bones and joints
  B. The ankle is the most common anatomic location of NTM tenosynovitis
  C. The clinical presentation for NTM and tuberculous osteoarticular infections is very similar
  D. Chemotherapy is unnecessary when surgical debridement is used to treat osteoarticular infections with NTM
3. Which of the following treatments is appropriately matched with its NTM skin or soft tissue infection?
  A. *Mycobacterium marinum*: Doxycycline monotherapy is acceptable for severe infection
  B. *Mycobacterium ulcerans*: Clarithromycin is the treatment of choice
  C. *M ulcerans*: Minocycline is the treatment of choice
  D. *M avium* complex: Treatment usually consists of 3 antibiotics for 6–12 months
4. Which of the following statements about rapidly growing mycobacteria (RGM) is most accurate?
  A. They respond to sterilization with formaldehyde solutions only
  B. They include organisms, such as *Mycobacterium fortuitum* and *Mycobacterium chelonae*
  C. Symptoms of RGM always occur within 4 weeks of exposure
  D. The usual duration of antibiotic therapy for infection with RGM is 1–2 months

### Activity Evaluation

**Table:** 

**1. The activity supported the learning objectives.**				
Strongly Disagree				Strongly Agree
1	2	3	4	5
**2. The material was organized clearly for learning to occur.**				
Strongly Disagree				Strongly Agree
1	2	3	4	5
**3. The content learned from this activity will impact my practice.**				
Strongly Disagree				Strongly Agree
1	2	3	4	5
**4. The activity was presented objectively and free of commercial bias.**				
Strongly Disagree				Strongly Agree
1	2	3	4	5

